# Exposure Models for REACH and Occupational Safety and Health Regulations

**DOI:** 10.3390/ijerph17020383

**Published:** 2020-01-07

**Authors:** John William Cherrie, Wouter Fransman, Gerardus Antonius Henrikus Heussen, Dorothea Koppisch, Keld Alstrup Jensen

**Affiliations:** 1 Centre for Human Exposure Science, Institute of Occupational Medicine, Edinburgh EH14 4AP, UK; 2 Institute of Biological Chemistry, Biophysics and Bioengineering, Heriot Watt University, Edinburgh EH14 4AS, UK; 3 Risk Analysis for Products in Development, TNO, 3700AJ Zeist, The Netherlands; Wouter.Fransman@tno.nl; 4 Cosanta BV, 1183 AS Amstelveen, The Netherlands; Henri.Heussen@Cosanta.nl; 5 Monitoring of Working Conditions, Institute for Occupational Safety and Health of the German Social Accident Insurance (IFA), 53757 Sankt Augustin, Germany; Dorothea.Koppisch@dguv.de; 6 The National Research Centre for the Working Environment, DK-2100 Copenhagen, Denmark; KAJ@nfa.dk

**Keywords:** ART, registration, evaluation, Authorisation and Restriction of Chemicals (REACH), Advanced REACH Tool, Stoffenmanager^®^, control banding

## Abstract

Model tools for estimating hazardous substance exposure are an accepted part of regulatory risk assessments in Europe, and models underpin control banding tools used to help manage chemicals in workplaces. Of necessity the models are simplified abstractions of real-life working situations that aim to capture the essence of the scenario to give estimates of actual exposures with an appropriate margin of safety. The basis for existing inhalation exposure assessment tools has recently been discussed by some scientists who have argued for the use of more complex models. In our opinion, the currently accepted tools are documented to be the most robust way for workplace health and safety practitioners and others to estimate inhalation exposure. However, we recognise that it is important to continue the scientific development of exposure modelling to further elaborate and improve the existing methodologies.

## 1. Introduction

Models are now widely used in regulatory risk assessments for industrial chemicals as well as in the management of these and other process that generate hazardous substances in workplaces. In Europe, the Registration, Evaluation, Authorization and Restriction of Chemicals (REACH) regulations allow registrants to rely on exposure estimates made using a variety of mathematical models, including the Targeted Risk Assessment (TRA) tool developed by the European Centre for Ecotoxicology and Toxicology of Chemicals [1], Stoffenmanager^®^ at www.stoffenmanager.com [2] and the Advanced REACH Tool (ART) [3]. In addition, models like ART and Stoffenmanager^®^ are in line with and are used under the EU health and safety regulations Directive 98/24/EC—risks related to chemical agents at work and Directive 2004/37/EC—carcinogens or mutagens at work. The development of models for this use has come a long way in the last 20 years. In the 1990s, the British Health and Safety Executive developed the Estimation and Assessment of Substance Exposure (EASE) tool to support risk assessment in Europe for new and priority existing substances, the regulations that preceded REACH [4]. However, early tools such as EASE were relatively crude and unreliable [5,6]. 

Because there are 2.5 billion workers around the world who have no access to health and safety professionals, control banding (CB) strategies have been developed and grown rapidly in recent years [7]. Such strategies can help prevent work-related illness and injury for those without professional support, and there are now CB tools for all types of hazardous chemicals, including new emerging risks like nanomaterials and for ergonomic hazards and injury prevention [8,9]. At the heart of all these tools is a simple concept or a more elaborate exposure model to rank or estimate the levels of exposure. Originally, CB was conceived as an action-oriented, qualitative risk assessment strategy, offering solutions and suggested control measures to users through “toolkits”. An early example of a CB tool is the British Control of Substances Hazardous to Health Regulations—COSHH-Essentials e-tool [10], which is still widely used and has formed the basis of many other CB tools. During the last decade, CB has gone beyond its traditional qualitative approach with some chemical CB tools, such as Stoffenmanager^®^ and the Einfaches Maßnahmenkonzept für Gefahrstoffe tool—EMKG-Expo-Tool, providing quantitative exposure assessments. Stoffenmanager^®^ therefore has many uses, as a CB tool, as an exposure assessment model in REACH and in helping ensure compliance with health and safety regulations. The tool was launched in 2003, before the REACH legislation came into force on 1 June 2007.

It is important that the regulatory models, as well as CB tools, can be used with a reasonable level of information demand and by a wide variety of assessors with varying degrees of knowledge and skills. In particular, CB tools need to be accessible to individuals with little or no scientific expertise, because these methods are intended to be used by health and safety practitioners and others at company level, including small and medium size enterprises. The tools must therefore have a balanced level of information requirements to be able to perform the exposure assessments through user-friendly interfaces, which lowers the risk of entry errors and user-variability in the assessment. Higher tier models can request a higher level of input information and in return provide an exposure estimate with reduced uncertainty.

Recently, a number of scientists have discussed the basis of mechanistic exposure models and questioned the acceptance of these tools for use in REACH and health and safety regulations [11]. In particular, they assert that the exposure modifying factors in these models are not always clearly described or correctly implemented. They argue that these model tools have poor predictive capability and that physical mass-balance models are the only appropriate way of modelling exposure for regulatory risk assessments. The aim of this paper is to address the key points raised in these discussions and to make the case for the applicability and continued use of the existing tools. 

## 2. Modelling of Exposures for Regulatory Risk Assessment Considering Variability of Exposure

It is clear that the availability of reliable and accurate exposure models is critical, as the European occupational hygiene community cannot collect sufficient numbers of exposure measurements to obtain exposure estimates for all relevant REACH scenarios or to comply with health and safety regulations. A tiered approach is proposed for REACH in which comparatively simple assessments are performed on all substances (Tier 1 assessment, e.g., using the TRA tool) with relatively high model performance uncertainty, followed by more elaborate evaluations for selected chemicals with higher associated risk (higher tier assessment, often referred as using Tier 1.5 or Tier 2 tools), which reduce the model uncertainty. Exposure concentrations vary substantially in any exposure scenario. This may result from differences across locations (e.g., different ventilation systems), workers (e.g., behaviour while handling chemicals) and time (e.g., time-activity patterns). These sources of variability may contribute to between-company, between-worker and within-worker variability. This variability in exposure, together with the model uncertainty should be taken into account and transparently provided in any exposure assessment model. The currently available exposure assessment models, such as Stoffenmanager^®^ and ART, take this variability and uncertainty into account by providing various percentiles of the exposure distribution and providing confidence intervals with these exposure estimates.

Modelling of exposures for regulatory risk assessment is a difficult task and presents different challenges than modelling of exposure levels in single specific work situations. Regulatory risk assessment models must be able to estimate exposure levels for loosely specified exposure scenarios where a substance will be used, or a process undertaken. An example could be, “use of paint A for industrial spraying in a workshop”. In such a case it is not possible to tightly specify the contextual conditions, such as the airborne release fraction from the spray process, the room size, the ventilation characteristics of the environment where the agent will be used or the specific design of exposure control measures that may be in place. All these factors would be needed for a good prediction using a mass-balanced model [12]. Other factors, such as worker position and behaviour also play an important role and may cause additional variation in exposure [13]. Under the REACH legislation it is necessary to show the safe use of a substance in exposure scenarios, which always represents an extensive group of workplaces. In the example above, the differences in important determinant parameters will vary considerable between different paint shops. Therefore, it is not enough to estimate the exposure at one specific workplace, but it is necessary to estimate possible exposures for all workplaces that are covered by the exposure scenario. This may be conducted in different ways with different assumptions. To meet this requirement, Stoffenmanager^®^ and ART calculate not only a point estimate, but a distribution of exposure circumstances across different specific workplaces within one exposure scenario. This is done by first calculating an average exposure level that describes the situation in general and then to include the variation in exposures measured in a range of specific workplaces. It is considered an advantage of these models that the variation in exposures actually found in workplaces is included into the modelling process [14]. The developers of the tools generally recommend using the 90th percentile of this distribution as a conservative estimate of the expected exposure at workplaces fulfilling the conditions of the exposure scenario. The tools therefore provide a balanced conservative overestimate of exposure for the class of situations covered by the given exposure scenario.

## 3. Exposure Model Structure and Exposure Modifiers

The Stoffenmanager^®^ and ART models for occupational exposure have a multiplicative form with individual modifying factors representing relative changes in exposure, these factors are generally referred to as “multipliers”. The tools were developed from conceptual source-receptor exposure models that were based on sound scientific principles [15]. Koivisto and colleagues [11] recently discussed these models, arguing that mathematically derived physical mass-balance models provide a stronger basis for case-specific exposure assessments. The mass-balance models are generally based on a mathematical solution of the equations governing the emission, transport, losses of substances from the air and ventilation air with or without local emission control [16]. These models may comprise one, two or more compartments with air and pollutants being exchanged between the different spaces. Mass-balance models are indeed conceptually more refined than the models used in regulatory risk assessment. However, to work well, these models require specific details of the scenario being modelled, including the mass emission rate from the source or sources, information that is not generally available for REACH exposure scenarios or even for specific individual workplaces. Certainly, knowing the emission rates and contextual parameters can make it possible to predict exposure or contaminant concentrations in specific situations relatively well by compartment modelling, e.g., papers by Ribalta et al. [17] and Jensen et al. [18]. However, even when all key information is known to a high degree, there can still be challenges in predicting the exposure levels accurately. For example, Ribalta et al. [19] used a single-compartment mass balance model of a thermal spray scenario, where the exposure was underestimated by between 30% and 80%, and the best predictions were achieved only after more advanced re-modelling. Some of these issues may be related to the correct determination of source strengths, which can be difficult to achieve. Jensen and colleagues [18], for example, showed a dramatic influence of sampling position for determination of source strengths in a controlled chamber test. Using dustiness as a surrogate for emission rate is also still associated with uncertainties, and there is a need for further understanding of the link between dustiness determined with specific test methods (e.g., EN15051; EN17199-1-4) and how the values from these different methods translate into an emission rate for a given work scenario. External parameters, such as storage conditions of powders, can have important effects on the dustiness and potential exposure [20]. Emission/source strength databases are still at an early stage and extensive work is needed to establish standards to produce harmonized release rate data and subsequently to generate sufficiently robust datasets for the required REACH process codes (PROCS) for general exposure assessment. The state-of-the-art and requirement for such future endeavours was discussed in Koivisto et al. [11,21].

In our opinion, the key question for modelling exposure to hazardous substances in occupational safety and health is not the model form, but whether the models provide appropriate results for the purpose and can be based on data available for users in the real world, a point that is recognized by Koivisto et al. [11]. In the case of mass-balance models it is of no help if the balance is correct, but the different sources and sinks involved in the mass-balance are not adequately known or estimated, e.g., a considerable error could already occur if the proportion of the emission that is leaving zone 1 in the case of a two-zone model is under or overestimated because of limited knowledge about the air exchange between model compartments—data generally not available for specific workplaces or for exposure scenarios in general. Only recently are research studies being undertaken to quantify air exchange relevant to multi-compartment modelling [22]. 

It has also recently been claimed that the “exposure modifying factors” of the currently accepted mechanistic REACH tools “are not always clearly described” and incorrectly calculated for general ventilation in Stoffenmanager^®^ and ART. Further, Koivisto and colleagues [23] identified errors in the calculation of the numeric values for the general ventilation multiplier originally formulated by Cherrie [24]. However, in a later paper [25] these multipliers were derived again, and these data were used for development of ART and on checking these data we find no errors. Koivisto et al. [23] incorrectly presents the differences between their calculations and those in the later paper [25], which were on average about 5% higher, a difference that we believe could reasonably be explained by small differences in the calculation methods. For Stoffenmanager^®^, the categorization of parameters and the allocation of scores for categories were partly taken from the work by Cherrie and colleagues [24,26], but were not directly translated into Stoffenmanager^®^ as Koivisto et al. [23] assumes. The scores for reduction by general ventilation both for near-field and far-field sources—dependent on room size—were modified to construct a simpler model as described in Marquart et al. [2] and Tielemans et al. [27]. Thus, Koivisto et al. [23] are not appropriately comparing the multiplier values actually used in ART and Stoffenmanager^®^ with their own multiplier calculations and the claim of error is unsubstantiated. 

In our opinion, the multipliers in ART and Stoffenmanager^®^ are clearly described. These models are extensively documented in peer-reviewed scientific papers and associate technical reports, which are available from the tool websites (https://stoffenmanager.com/what-is-stoffenmanager/; https://advancedreachtool.com/science.aspx). However, we fully accept that it is necessary to categorise many of the tool inputs for practical regulatory models and that this may be perceived as the parameter being loosely defined. For example, for dustiness of solids in ART there are five categories, from “firm granules, flakes or pellets” through to “extremely fine and light powder”. These categories and their associated weights in the tool are based on experimental evidence [3], but details of dustiness of individual products evaluated under REACH are generally still not available, which necessitates the use of categories in the tools. We accept that it can be difficult to assign specific categories in practice when using tools, for example when is a product a “fine dust” or an “extremely fine” dust? However, this is not a drawback of the model tools, but rather a limitation of the data currently available in regulatory risk assessments. New developments of standard dustiness tests (EN15051:2013; EN17199:2019) and recent inclusion of dustiness data as an information requirement for nano-substance registration in REACH, will hopefully improve this situation, and in the future, it may be possible to include such data for all powder materials.

## 4. Calibration and Validation of the Tools

The mechanistic modelling that underpins both Stoffenmanager^®^ and ART are based on a theoretical analysis in which the multipliers were derived based on physical laws and data in combination with expert judgement. The model tools were subsequently calibrated using measurement data (often considered as being the “gold standard” in occupational exposure assessment). So even if there were any minor errors made in the numerical weights for the dispersion multiplier, this will have been compensated for in the calibration with quantitative estimates of exposure, with more than 3000 exposure measurement data for ART and almost 1000 measurements for Stoffenmanager^®^ [27,28]. This calibration process not only accounts for the uncertainties associated with the multipliers and the model uncertainty in the average model output (which is inherent to any model), but also transparently indicates the level of confidence in the model output, and enables the model user to select the level of conservatism and confidence they wish.

In addition, after this calibration of the ART model and Stoffenmanager^®^, several validation studies with independent sets of measured data from the EU, USA, Taiwan and Korea have been published in peer reviewed literature. For example, the validation of Stoffenmanager^®^ is now based on almost 7000 measurements, which represents an impressive scientific effort. From a total of 21 studies on external validation, sensitivity and robustness, Spinazzè et al. [29] concluded that Stoffenmanager^®^ tends to overestimate low exposures and underestimate high exposures, but at the same time—by using the recommended 90th percentile—generally guarantees conservative estimates and shows a high degree of robustness. They judged Stoffenmanager^®^ to be the most robust model for REACH and ART to be the most accurate and precise model, with a medium amount of conservatism. However, one exception on the conservatism was seen for Stoffenmanager^®^ for exposure to low volatile substances, released as a result of outdoor spraying activities without local exhaust ventilation (aerosol formation: PROC7 and PROC11 in REACH), which was underestimated [30]. For these activities, the Stoffenmanager^®^ tool owner recommends using the 95th percentile estimation, or to comply with a risk characterization ratio (RCR) = 0.5 (instead of RCR = 1). 

Until now, we have not seen any publications that transparently describe mass-balanced modelling for chemical regulatory risk assessment, including the parameter uncertainties and assumptions that have to be made, although some progress is being made in developing models for the more restricted case of nanomaterials, for example work by Ribalta et al. [17] and Belut et al. [31]. Also, we have not been able to identify any validation studies demonstrating that mass-balance modelling gives results that perform better for exposure assessment at scenario level than the currently available model tools for REACH. Generation of high-quality measurement data is highly warranted to allow such tests and comparisons for further improvement of regulatory risk assessment. Also, we consider that there needs to be an extensive expansion in the availability of emission rate data, control efficacy levels, contextual and behavioural parameters and specific hard-coded model parameters.

## 5. Conclusions

We believe that those who criticize the currently recommended REACH tools have underestimated the difficulty in exposure modelling within a regulatory framework or the practical use of CB tools [21]. The comments tend to focus on what is possible by expert researchers and not what is practicable and possible for health and safety practitioners. To enable application of more advanced models as a basis for CB and regulatory risk assessment, one also needs to work to ensure good data availability, applicability, competences, recognition and acceptance of tools by all relevant stakeholders. It may be expected that in principle, REACH tools may provide lower accuracy in the estimation of exposure than more refined mass-balance models, but higher tier model tools can result in greater user variability. A model on its own, either mass balanced or mechanistic, will not make any difference in daily occupational health and safety practice, implementation is key. In a previous study, for example, it was shown that providing interactive user support was necessary for successful implementation of Stoffenmanager^®^ [32]. 

It has also been suggested that we are unwilling to open the currently accepted models to external scientific scrutiny [23]. This is not the case; the models have been published in peer reviewed literature and are freely available online with sufficient guidance material provided to guide the user in making the correct selections for model inputs. Many researchers, companies and regulators have found their way to using and supporting these modelling tools, and many of them have performed validation studies to provide evidence of their usefulness [27]. We welcome and participate in the development and use of different modelling approaches, including mathematically derived physical mass-balance modelling. In our opinion, it should be recognised that extensive research is needed to define useful quantitative values for the key model parameters and their variability. This includes, not least, the establishment of elaborate source emission strength databases for a large number of substances and work scenarios. The common goal should be the development of different complementary approaches for exposure and risk assessment that can contribute to establish healthier working environments for people working with hazardous chemicals.
